# Safranal exerts a neuroprotective effect on Parkinson’s disease with suppression of NLRP3 inflammation activation

**DOI:** 10.1007/s11033-024-09537-y

**Published:** 2024-04-29

**Authors:** Wenping Yang, Yongyue Wei, Jin Sun, Caixia Yao, Fen Ai, Haixia Ding

**Affiliations:** 1 Division of Neurology, Department of Geriatrics, Jiangsu Province Hospital and Nanjing Medical University First Affiliated Hospital, NO. 300 Guangzhou Road, Nanjing, China; 2 Department of Biostatistics, School of Public Health, Nanjing Medical University, Nanjing, China; 3 Department of Nuclear Medicine, Jiangsu Province Hospital and Nanjing Medical University First Affiliated Hospital, Nanjing, China; 4 Departement of Endocrine, Nanjing Gao Chun People’s Hospital, Nanjing, China; 5 Department of Emergency, Tongji Medical College, The Central Hospital of Wuhan, Huazhong University of Science and Technology, NO. 26 Shengli Street, Wuhan, Jiang’an District China

**Keywords:** Safranal, Parkinson’s disease, NLRP3, Neuroprotective

## Abstract

**Background:**

Parkinson’s disease (PD) is a common central nervous system neurodegenerative disease. Neuroinflammation is one of the significant neuropathological hallmarks. As a traditional Chinese medicine, Safranal exerts anti-inflammatory effects in various diseases, however, whether it plays a similar effect on PD is still unclear. The study was to investigate the effects and mechanism of Safranal on PD.

**Methods:**

The PD mouse model was established by 1-Methyl-4-phenyl-1,2,3,6-tetrahydropyridine MPTP firstly. Next, the degree of muscle stiffness, neuromuscular function, motor retardation and motor coordination ability were examined by observing and testing mouse movement behavior. Immunofluorescence staining was used to observe the expression of tyrosine hydroxylase (TH). The dopamine (DA) content of the striatum was detected by High-performance liquid chromatography (HPLC). The expression of TH and NLRP3 inflammasome-related markers NLRP3, IL-1β, and Capase-1 were detected by Real-time Polymerase Chain Reaction (qRT-PCR) and western blotting (WB) respectively.

**Results:**

Through behavioral testing, Parkinson’s mouse showed a higher muscle stiffness and neuromuscular tension, a more motor retardation and activity disorders, together with a worse motor coordination compared with sham group. Simultaneously, DA content and TH expression in the striatum were decreased. However, after using Safranal treatment, the above pathological symptoms of Parkinson’s mouse all improved compared with Safranal untreated group, the DA content and TH expression were also increased to varying degrees. Surprisingly, it observed a suppression of NLRP3 inflammation in the striatum of Parkinson’s mouse.

**Conclusions:**

Safranal played a neuroprotective effect on the Parkinson’s disease and its mechanism was related to the inhibition of NLRP3 inflammasome activation.

## Introduction

Parkinson’s disease (PD) is a common neurodegenerative disease of the central nervous system in which the selective loss of dopamine (DA) neurons in the substantia nigra leads to the exhaustion of DA released from the nerve endings of the striatum [1, 2], which causes clinical motor symptoms that mainly manifest as resting tremors, muscle stiffness, and slow movement [3, 4]. Moreover, PD is accompanied by nonmotor symptoms, such as hypoosmia, gastrointestinal dysfunction, cardiovascular dysfunction, sleep disturbance and neurocognitive dysfunction [5].

Neuroinflammation is one of the significant neuropathological hallmarks of PD, and it exacerbates dopaminergic neuronal degeneration in the substantia nigra [6, 7]. The nucleotide-binding oligomerization domain-like receptor protein 3 (NLRP3) inflammasome is a multimolecular complex located in the cytoplasm that controls the processing, maturation and release of the cytokines NLRP3, IL-1β and Capase-1 [8]. This inflammasome is expressed in epithelial cells, granulocytes, and monocytes, and it can be activated by a variety of model molecules with different morphological structures and molecular sequences [9, 10]. In addition, the NLRP3 inflammasome plays a pivotal role in neuroinflammation in PD [11, 12]. Linfang Chen et al. demonstrated for the first time that inhibition of NLRP3 inflammasomes can reduce the neuroinflammation of astrocytes in MPTP-induced PD mouse models [13]. Wei Wang et al. found that inhibiting the NLRP3 inflammasome in an MPTP-induced PD mouse model effectively reduced neuronal damage in microglia [14]. Xiaofei Qiu et al. showed that inhibiting NLRP3 inflammasome overactivation can protect dopaminergic neurons in mouse PD models [15], while Shuxuan Huang et al. indicated that NLRP3, rather than NLRP1 and NLRP2, may represent a key inflammasome that promotes the pathogenesis of MPTP-induced PD [16]. These findings implied that the NLRP3 inflammasome might be an important substance for related neurodegeneration in PD.

Safranal is a natural aromatic compound derived from Crocus sativus, and it is responsible for imparting the characteristic aroma to saffron [17]. Safranal is a pharmacologically active compound that possesses anticonvulsant and antidepressant properties, and its numerous biological activities, such as anti-inflammatory, antioxidative stress, antiapoptotic, and anticancer activities, have been well explored [18]. Chen Zhang et al. suggested that Safranal promotes the recovery of neuronal function in rats and the effect is related to its anti-apoptotic, anti-inflammatory, and edema-attenuating effects [19]. Mehak Gupta et al. showed that Safranal can inhibit the inflammatory response mediated by NLRP3 inflammasomes and can be used treat chronic inflammatory diseases involving the activation of NLRP3 inflammasomes [20]. However, it is still unclear whether Safranal has a therapeutic effect on Parkinson’s disease.

In order to further clarify the role of Safranal in Parkinson’s disease and its related mechanisms, the Parkinson’s mouse model was constructed firstly. The study mainly explored whether Safranal improved the pathological symptoms of Parkinson’s disease mouse, increased the expression of TH and DA content in Striatum, to clarified the relevant mechanisms, which provide a new treatment way for clinical Parkinson’s disease.

## Materials and methods

### Animals

C57BL male mouse were purchased from the Laboratory Animal Center of Nanjing Medical University (Nanjing, China) and housed at room temperature (22–25 °C) under a 12 h light/12 h dark cycle. Animal experiments followed the animal management regulations of Jiangsu Province Hospital (First Affiliated Hospital of Nanjing Medical University) and guidelines for animal care and use from the National Institutes of Health (NIH; Bethesda, MD, USA). The ethical approval protocol number is ZDRCA2016001. Eight animals were included per group. After adapting to the environment for 1 week, all mice underwent 3-day motor function training, followed by 1-Methyl-4-phenyl-1,2,3,6-tetrahydropyridine (MPTP) to build an in vivo model of Parkinson’s disease [21]. MPTP-HCL was subcutaneously administered at a dose of 25 mg/kg body weight per day for 5 days. Based on the drug instructions and previous relevant studies [18, 19], after we conducted preliminary experiments using different concentrations of Safranal, it was injected intraperitoneally in volumes of 0.1, 0.2 and 0.4 ml/kg (Density (d) = 0.98 g/ml; Molecular weight (Mw) = 150.22) using a Hamilton syringe for 14 days.

### Behavioral test

Before the start of the experiment, all experimental mice received stable baseline performance training to eliminate experimental errors, and mouse that did not meet the conditions were removed. A catalepsy test was used to detect the degree of muscle stiffness. In short, hind limbs of the mouse were placed on a wooden block (5 cm high), and the time required for the hind limbs to move to the ground was recorded [22]. In the grip strength experiment, evaluate the neuromuscular function, which was recorded in units of gmf [23]. In the rotarod test experiment, the mice were kept on the rotating rod in an acceleration mode of 4 to 45 rpm, with a cutoff time of 5 min. The fall time was automatically recorded by the sensor [24]. The pole test was used to measure the degree of motor retardation. Specifically, the mice were allowed to run along a vertical rod (50 cm long), and the time required to reach the ground was recorded [25]. In the walking track analysis experiment, the paws of the mice were painted with nontoxic ink, the animals were allowed to walk freely on white paper, and the distance between two consecutive paw prints was recorded [26]. In the open field test (OFT) experiment, the mice were placed in a wooden box (50 cm^3^) in the central square and behavioral activities were recorded by ANY-maze behavior tracking software (version 5.0). The total distance traveled, exercise time, average speed and maximum speed were recorded [27].

### High-performance liquid chromatography (HPLC)

HPLC was used to detect the level of DA in tissue. The mice fasted for 12 h and were euthanized using cervical dislocation method. Midbrain and Striatum were taken according to the stereotactic map of mouse brain. Then, Striatum weighed and homogenized in 0.1 mol/l HClO4, incubated on ice for 1 h, and centrifuged for 20 min (4 °C, 12,000 r). Then, the supernatant was obtained and mixed with the HPLC solution (consisting of 63.5 mM citric acid monohydrate, 60.9 mM dehydrated trisodium citrate, 0.1 M EDTA and 0.5 M sodium 1-decane sulfonate). The pH value was adjusted to 4.3, and the above mixture was injected into the chromatographic column. The data are recorded in pg dopamine/mg tissue.

### Immunofluorescence staining

Slices of mouse striatal tissue were blocked with 5% goat serum (ImmunoReagents, USA), and incubated with primary antibodies against TH (1:500, Santa Cruz) overnight at 4 ◦C. Then, Alexa Fluor 568-conjugated goat anti-rabbit IgG (1:1000, Abcam) and Alexa Fluor 488-conjugated goat anti-mouse IgG (1:1000, Invitrogen) were incubated at room temperature for 1 h. Next, sections were stained with 4′,6-diamidino-2′ -phenylindole (Sigma, USA). Finally, the fluorescence changes were observed through a fluorescence microscope, green fluorescence intensity represents the expression level of TH- positive cells (Olympus, Japan).

### Real-time polymerase chain reaction (qRT–PCR)

First, PCR primers were designed with Primer Premier 5.0 and dissolved to a concentration of 10 mM. Second, the substantia nigra of the midbrain was collected, total RNA was extracted and isolated using the TRIzol method, and the RNA concentration was measured. Then, a cDNA synthesis kit was used to convert RNA to cDNA. DNA strands complementary to the template were synthesized through DNA denaturation (90–96 °C), annealing (25–65 °C), and extension (70–75 °C). The cycle times were 25 to 30 times, and the Tm value was 4(G + C) + 2(A + T). Glyceraldehyde-3-phosphate dehydrogenase (GAPDH) was used as the internal control, and the relative gene expression levels were calculated by the 2^− ΔΔCt^ method. The primers for RT-PCR are shown in Table 1.

**Table 1:** Primers of RT-PCR

RNA	Sequences (F, forward; R, reverse)
TH	F: 5′-GATTGCTACCTGGAAGGAGGT-3′, R: 5′-AGTCCAATGTCCTGGGAGAAC-3’
NLRP3	F: 5’-GAGTTCTTCGCTGCTATGT-3′, R: 5’-ACCTTCACGTCTCGGTTC-3′
IL-1β	F: 5′-TTCAAATCTCACAGCAGCAT-3′, R: 5′-CACGGGCAAGACATAGGTAG-3′
Capase-1	F: 5′-GGCCCAGGAACAATGGCTGC-3′, R: 5′-GGGTCACAGCCAGTCCTCTTA-3′
GAPDH	F: 5′-AGAGGCAGGGATGTTCTG-3′, R: 5′-GACTCATGACCACAGTCC ATGC-3’

### Western blotting

The substantia nigra tissue was cut it into small pieces, and then 400 µL of single detergent lysis solution (including PMSF) was added. The solution was lysed for 30 min and centrifuged at 12,000 rpm at 4 °C for 5 min, and the supernatant was then collected. The concentration of the protein to be tested was measured and recorded and SDS–PAGE electrophoresis (voltage 40 V) was performed. The proteins were transferred to a membrane, which was stained with 1× Ponceau staining solution for 5 min, moved to 5% skim milk, sealed for 2 h, incubated with the primary antibody and horseradish peroxidase (HRP)-conjugated secondary antibody (1:5000; Cell Signaling Technology, Danvers, MA) for 2 h at room temperature, and finally subjected to chemiluminescence, development, and fixation. Following primary antibodies were used: anti-TH (1:1000; Sigma, T2928), anti-NLRP3 (1: 1000, BioVision, A1767-100), anti-IL-1β (1:1000; Sigma, I-3767); anti-Capase-1 (1:1000; Sino Biological, 90,011-MM02) anti-GAPDH (1:1000, Sino Biological, MB10094-T52).

### Statistical analysis

Data analysis was conducted by professionals who do not understand the grouping of experiments. All values were expressed as Mean ± Standard deviation (SD). Differences among different groups were compared by one-way analysis of variance (ANOVA) using the GraphPad software. A two-tailed Student’s t-test was used to compare the two groups. *P* < 0.05.

## Results

### The Parkinson’s mouse model was constructed successfully using the MPTP- induced method

**Fig. 1:**
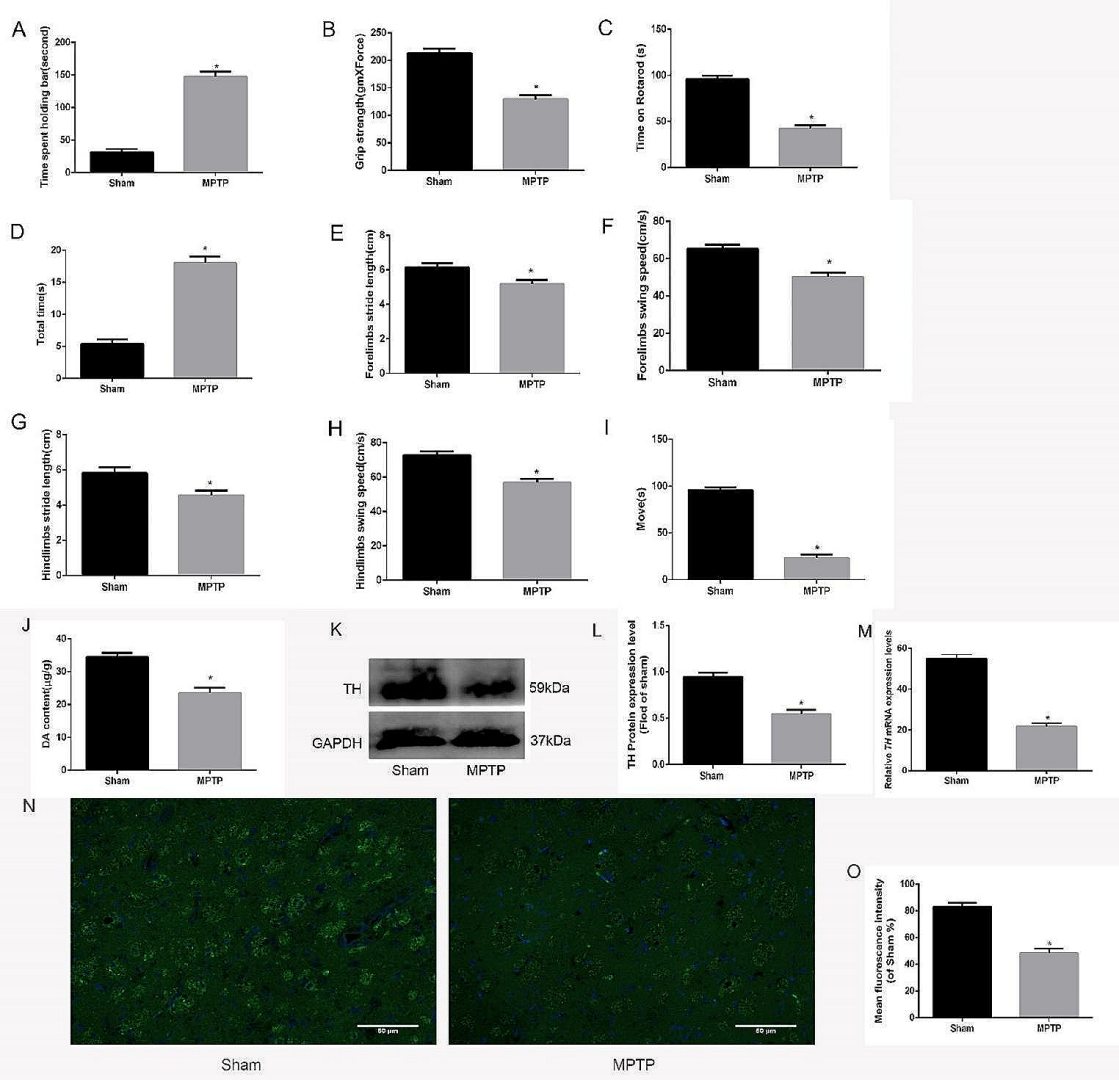
The Parkinson’s mouse model was successfully constructed using the MPTP method. **A** Stiffness was assessed by the Catalepsy test. The graph shows the time of the movement of the hind limb to the surface. **B** Neuromuscular tension is tested through grip strength. **C** Passive movement was assessed with a rotarod, and the graph shows the residence time on the rotating rod. **D** Adaptive exercise was assessed with a pole climbing test, and the graph shows the time to turn around and climb down. **E-H** Walking track analysis of the swing speed and stride length of the forelimbs and hindlimbs. **I** Spontaneous movement was assessed with the open field test, and the graph shows the movement time (*n* = 8, ^*^P < 0.05). **J** Dopamine (DA) content in the striatum in the Sham and MPTP-treated groups. **K** TH (tyrosine hydroxylase) protein expression in the striatum in the Sham and MPTP-treated groups. **L** Quantitative analysis of TH protein expression. **M** mRNA expression of *TH* in the striatum in the Sham and MPTP-treated groups. **N** Immunofluorescence staining of Striatum in two groups of mice. The magnification is 200 times, and the scale is 50 μm. **O** The mean fluorescence density analysis (*n* = 6, ^*^P < 0.05)

The experiment was divided into the MPTP treatment group (abbreviated as MPTP) and the Sham group (abbreviated as Sham), and eight mice included in each group. In order to evaluate whether the Parkinson’s model was successfully constructed, behavioral testing of Parkinson’s was performed, including the catalepsy test, grip strength, rotarod test, pole test, walking track analysis, and open field test (OFT). Higher results were obtained for the catalepsy test(Fig. 1A) and pole climbing(Fig. 1D), while lower results were obtained for the grip strength(Fig. 1B), rotarod test(Fig. 1C), walking track analysis (including forelimb swing speed, hindlimb swing speed, forelimb stride length, hindlimb stride length) (Fig. 1E, F, G, H), and open field test (OFT) (Fig. 1I) in the MPTP-induced group compared with the Sham group, and the difference was statistically significant (*n* = 8, ^*^P < 0.05).

In order to further explore the pathological changes of Parkinson’s mouse, the DA content was detected by the HPLC and the mRNA and protein expression of TH were detected by PCR and WB. The results showed that the DA content and the TH expression in the MPTP group were both reduced compared with that in the sham group (Fig. 1J, K, L, M), and the difference was statistically significant (*n* = 6, ^*^P < 0.05). Finally, in order to further explore the changes of TH expression in the two groups, immunofluorescence staining of the striatum and the mean fluorescence density was performed through a fluorescence microscope, green fluorescence intensity represents the expression level of TH, the results showed that the green fluorescence in the experimental group was slightly lower than that in the Sham group (Fig. 1N, O), and the difference was statistically significant (*n* = 6, ^*^P < 0.05), which indicating a decrease of TH expression in the experimental group. Therefore, the Parkinson’s disease model was successfully constructed.

### NLRP3 inflammasome activation increased in Parkinson’s mouse model

**Fig. 2:**
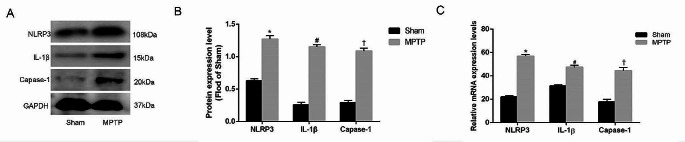
The expression of the NLRP inflammasome-related markers NLRP3, IL-1β, and Capase-1 in the Striatum. **A** NLRP3, IL-1β, and Capase-1 protein expression. **B** Quantitative analysis of NLRP3, IL-1β, and Capase-1 protein expression. **C***NLRP3, IL-1β*, and *Capase-1* mRNA expression (*n* = 6, ^*^P < 0.05, ^#^*P* < 0.05, ^†^*P* < 0.05). Data are expressed as the mean ± SD

In order to further observe the pathological mechanism of Parkinson’s mouse, NLRP3 related apoptosis were explored firstly. PCR and WB were performed to detect the expression of the NLRP3 inflammasome-related markers NLRP3, IL-1β, and Capase-1 in the striatum. The results showed that the protein expression of NLRP3, IL-1β, and Capase-1 increased in the MPTP group compared with the Sham group (Fig. 2A, B), and the difference was statistically significant (*n* = 6, ^*^P < 0.05). Similarly, the mRNA expression of *NLRP3, IL-1β*, and *Capase-1* also increased in the MPTP group compared with the Sham group (Fig. 2C), and the difference was statistically significant (*n* = 6, ^*^P < 0.05).

### Safranal ameliorated motor deficits of the Parkinson’s mouse

To determine the therapeutic effect of Safranal on Parkinson’s mouse, the mice were divided into Sham group, MPTP induced group, and Safranal treatment group. According to the different concentrations of Safranal treatment, the Safranal treatment group was further divided into low dose (0.1 ml/kg), medium dose (0.2 ml/kg), and high dose (0.4 ml/kg) groups.

**Fig. 3:**
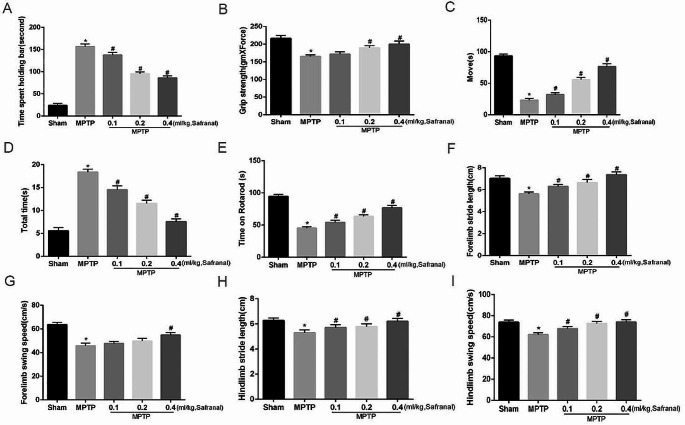
Safranal ameliorated motor deficits of the Parkinson’s mouse **A** Stiffness was assessed by the Catalepsy test, and the graph shows the time of the movement of the hind limb to the surface. **B** Neuromuscular tension is tested through grip strength. **C** Spontaneous movement was assessed with the open field test. The graph shows the movement time. **D** Adaptive exercise was assessed with the pole climbing test, and the graph shows the time to turn around and climb down. **E** Passive movement was assessed with a rotarod, and the graph shows the residence time on the rotating rod. **F, G, H, I** Walking track analysis of the swing speed and stride length of the forelimbs and hindlimbs. Data are expressed as the Mean ± SD. (*n* = 8, ^*^P < 0.05, ^#^*P* < 0.05)

In the catalepsy test, the graph shows the time of the movement of the hind limb to the surface. The results showed that in the Safranal treatment group, the movement time of the hind limb to the surface was shorter than that of MPTP induced group, and the difference was statistically significant (Fig. 3A, *n* = 8, ^*^P < 0.05, ^#^*P* < 0.05). Thus, Safranal improved the symptoms of limb stiffness of Parkinson’s mouse.

In the grip strength test, the results showed that the grip strength of mice in the Safranal treatment groups (0.2 and 0.4 ml/kg) increased compared with that of the MPTP group, and the effect was more obvious with increases of Safranal concentration, the difference was statistically significant (Fig. 3B, *n* = 8, ^*^P < 0.05, ^#^*P* < 0.05). Thus, Safranal enhanced the neuromuscular tension of Parkinson’s disease mouse.

In the open field test (Fig. 3C) and walking track analysis (Fig. 3F, G, H, I), the Safranal treatment groups (0.1, 0.2, and 0.4 ml/kg) showed increased exercise time, forelimb swing speed, hindlimb swing speed, forelimb swing speed, forelimb stride length, and hindlimb stride length compared with those of the MPTP-treated group, and the differences were statistically significant (*n* = 8, ^*^P < 0.05, ^#^*P* < 0.05). Thus, Safranal improved the spontaneous activity of Parkinson’s disease mouse.

In the pole climbing test (Fig. 3D), Safranal treatment group (0.1, 0.2, and 0.4 ml/kg) showed reduced the time of the mice spent turning around and climbing down, and the differences were statistically significant (*n* = 8, ^*^P < 0.05, ^#^*P* < 0.05). In the rotarod test (Fig. 3E), Safranal treatment group (0.1, 0.2, and 0.4 ml/kg) showed increased the time the mice stayed on the rotating rod, and the differences were statistically significant (*n* = 8, ^*^P < 0.05, ^#^*P* < 0.05). which indicating improvements in their passive movement and adaptive behavior.

In the study, the pole climbing test and rotarod test reflect the motor coordination ability while the pole climbing test and open field test reflect the motor retardation level. Therefore, above results indicated that Safranal improved the body stiffness, neuromuscular tension, coordination ability, and motor retardation of Parkinson’s disease mouse.

### Safranal increased the DA content and TH expression in Parkinson’s disease mouse

**Fig. 4:**
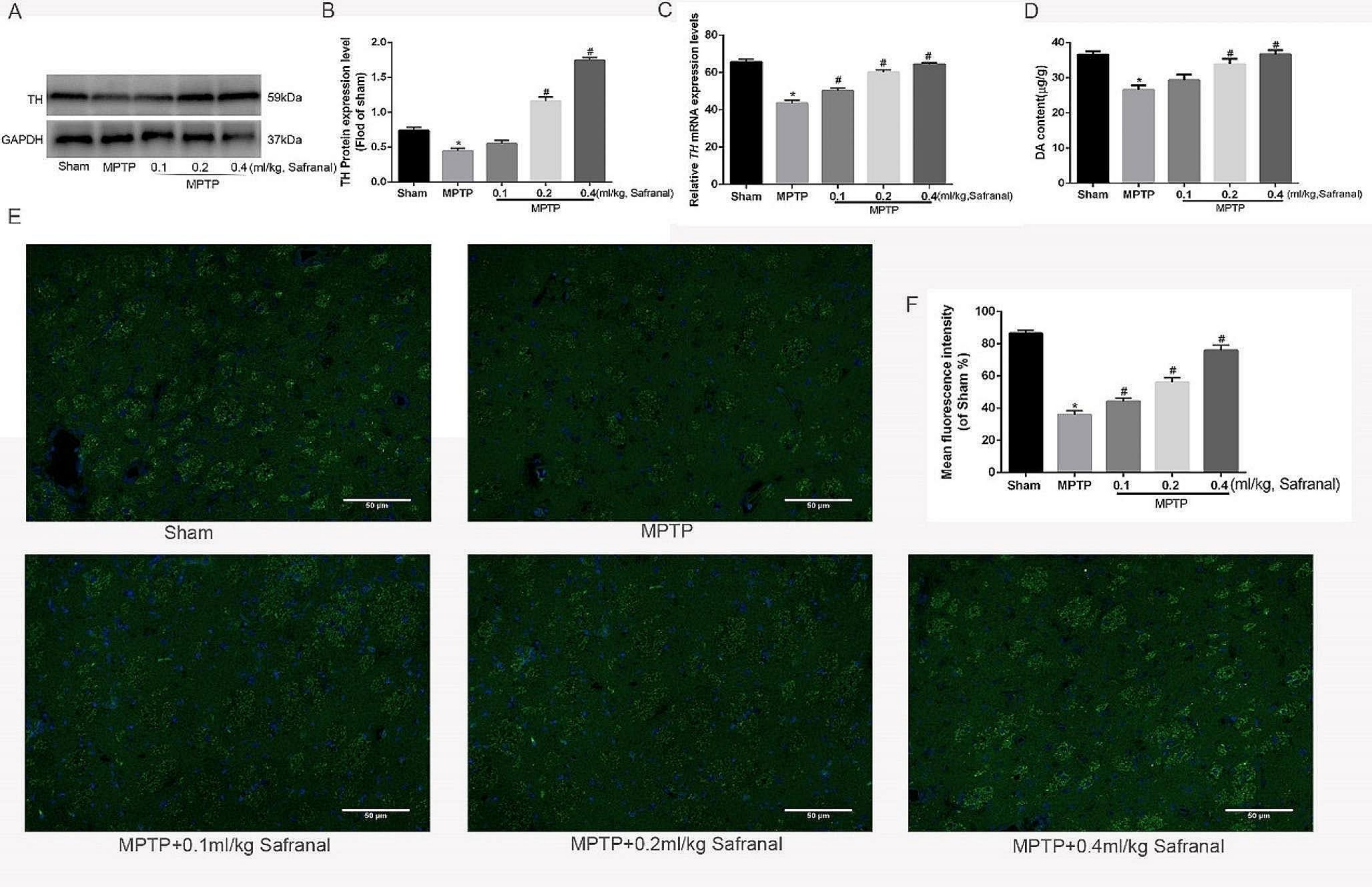
Safranal increased the DA and TH content in the Parkinson’s disease mouse **A** TH protein expression in each group. **B** Quantitative analysis of TH protein expression. **C** the mRNA expression of *TH* in each group. **D** DA content in each group. **E, F** Immunofluorescence staining of Striatum in each group. The magnification is 200 times, and the scale is 50 μm. Data are expressed as the Mean ± SD. (*n* = 6, ^*^P < 0.05, ^#^*P* < 0.05)

In order to further investigate the effect of Safranal on pathological changes in Parkinson’s mouse, the content of DA and the expression of TH in the striatum were detected. The results showed that 0.2 and 0.4 ml/kg Safranal increased TH protein expression while 0.1 ml/kg Safranal had no obvious effect. Statistically significant effects were only observed when the Safranal concentration reached 0.2 ml/kg, and the effect becomes more obvious as the drug concentration increases (Fig. 4A, B, *n* = 6, ^*^P < 0.05, ^#^*P* < 0.05). Simultaneously, Safranal (0.1, 0.2, and 0.4 ml/kg) increased TH mRNA expression, the difference was statistically significant ((Fig. 4C, *n* = 6, ^*^P < 0.05, ^#^*P* < 0.05). 0.2 and 0.4 ml/kg Safranal increased the DA content, and the effects were only observed when the Safranal concentration reached 0.2 ml/kg (Fig. 4D, *n* = 6, ^*^P < 0.05, ^#^*P* < 0.05).

Next, immunofluorescence staining of the Striatum was performed and the fluorescence changes were observed through a fluorescence microscope, green fluorescence intensity represents the expression level of TH, the results showed that compared with the MPTP induced group, The green fluorescence was higher than that in the Safranal treatment group (Fig. 4E, F), and the difference was statistically significant (*n* = 6, ^*^P < 0.05), which indicating an increase of TH expression in the Safranal treatment group. In summary, Safranal increased the expression of TH and DA content in Parkinson’s mouse.

### NLRP3 inflammasome were inhibited after using Safranal treatment in Parkinson’s disease mouse

**Fig. 5:**
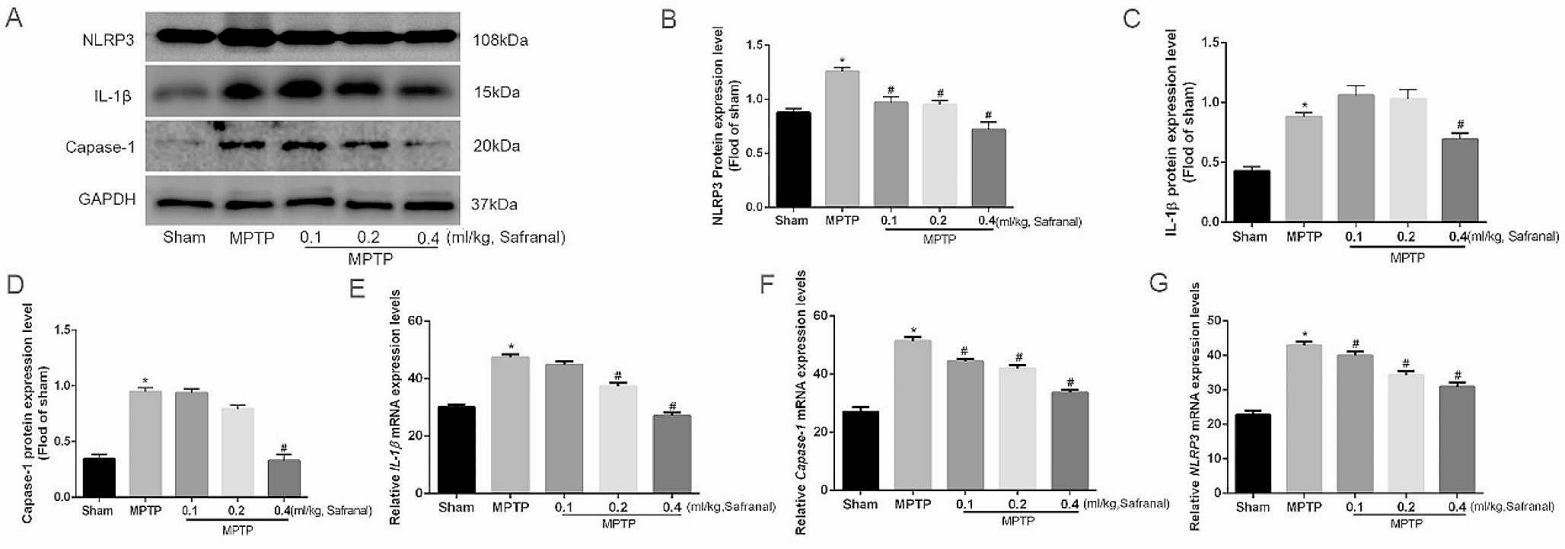
NLRP3 inflammasome were inhibited after using Safranal treatment in Parkinson’s disease mouse. **A** NLRP3, IL-1β, and Capase-1 protein expression level. **B** Quantitative analysis of NLRP3 protein expression level. **C** Quantitative analysis of IL-1β protein expression level. **D** Quantitative analysis of Capase-1 protein expression. **E***IL-1β* mRNA expression level. **F***Capase-1* mRNA expression level. **G***NLRP3* mRNA expression level. Data are expressed as the mean ± SD. (*n* = 6, ^*^P < 0.05, ^#^*P* < 0.05)

To explore the specific mechanism of the protective effect of Safranal on Parkinson’s mouse, different concentrations of Safranal were used in the Parkinson’s mouse in vivo. Then the expression of the NLRP3 inflammasome marker molecules NLRP3, IL-1β, and Capase-1 were detected. The experiments showed that after using different concentrations of Safranal (0.1 ml/kg, 0.2 ml/kg, and 0.4 ml/kg) in MPTP induced mice, the protein and mRNA expression of NLRP3, IL-1β, and Capase-1 were reduced (Fig. 5A-G), and the difference was statistically significant (*n* = 6, ^*^P < 0.05, ^#^*P* < 0.05). That is, NLRP3 inflammasome was suppressed in the substantia nigra after using Safranal treatment, and this inhibitory effect gradually strengthens with the Safranal concentration increases. Then it can be confirmed the NLRP3 inflammation suppression play a key function of Safranal in Parkinson’s disease. Safranal protected the Parkinson’s disease mouse via inhibition of NLRP3 inflammasome activation.

## Discussion

With a global prevalence of more than 6 million individuals and the incidence rate increase 2·5-times in prevalence over the past 30 years, Parkinson’s disease becomes the second most common neurodegenerative disease [28, 29]. As a degenerative disease of the central nervous system, the clinical symptoms of Parkinson’s disease mainly include static tremors, muscle rigidity, motor delay, posture balance disorders, etc. The motor disturbances cause progressive disability, with impairment in activities of daily living and reduced quality of life [30], which making Parkinson’s disease one of the leading causes of neurological disability and seriously affect the patient’s quality of life [5, 31]. Therefore, actively seeking new therapeutic drugs of Parkinson’s disease is of great clinical significance.

In this study, the Parkinson’s disease mouse was constructed by MPTP-induced ways [32]. It should be noted that the catalepsy test reflected the limb stiffness of mice, the grip strength reflected the neuromuscular tension, the rotarod test and the pole test signified the movement coordination ability, the walking track analysis and open field test represented the spontaneous motor activity, the pole test and open field test indicated the degree of decreased movement. Through the detection of behavioral tests of mice, ultimately, the MPTP-induced mice showed that the limb stiffness, the neuromuscular tension and the motor retardation increased, meanwhile, the motor coordination and the spontaneous motor activity impaired. which were consistent with the movement behavior patterns of Parkinson’s patients observed clinically. Besides, the content of DA and the expression of TH both reduced in striatum of brain in MPTP treated mice, which indicated that the Parkinson’s mouse model was constructed successfully.

As a traditional Chinese medicine, Safranal has brought researchers over the world to explore more effects of it for human health. Research on Safranal mainly focused on anti-inflammatory [33], antidepressant [17], anxiolytic [34], antiasthamatic [17], antihypertensive [35], anticancer [36] and so on. However, there is still relatively little research on the nervous system of Safranal. Research has shown that Safranal obtained from Saffron flower which offers a large number of neuroprotective actions [37]. Potential role of Safranal as neuroprotective agent has also been studied in an inflammation-associated neuronal apoptosis model where Safranal promoted the recovery of neuronal cells after Spinal cord injury [38]. And another study suggested that Safranal administration may prevent behavioral alterations by modulating the brain oxidative response in rats subjected to chronic restraint stress procedures [19].

To further elucidate the effect of Safranal on Parkinson’s mouse, different concentrations of Safranal were injected into Parkinson’s mouse intraperitoneally, it was found that the DA contents and TH expression in the striatum increased after Safranal treatment. Meanwhile, the limb stiffness relieved, neuromuscular tension improved, and motor coordination of adaptive, passive exercise and spontaneous exercise all ameliorated. The above results indicated that Safranal alleviated the symptoms of motor disorders in Parkinson’s mouse and it may be used to treat Parkinson’s disease. Moreover, there have been two previous studies on Safranal and Parkinson’s disease, one of which was Yi Zhao et al. demonstrated that Safranal promoted the production of functional DA cells and alleviated PD through in vitro and in vivo rat models [39], the other was P-K Pan et al., indicated that Safranal protected against rotenone-induced neurotoxicity associated with Nrf2 signaling pathway in vitro model of PD [40]. Surprisingly, their research was consistent with our research.

Moreover, we concluded that NLRP3 inflammasome play a key role in Safranal- afforded neuroprotective in PD mice. And Safranal played a neuroprotective effect on the Parkinson’s disease mouse and its mechanism may be related to the inhibition of NLRP3 inflammasome activation. Previous studies have shown that inhibiting the NLRP3 inflammasome in rats can have an antineurological effect [41], and inhibiting the activation of NLRP3 inflammasomes could reduce neuroinflammation during intracerebral hemorrhage [42]. This was also consistent with our research.

In this study, the behavioral changes in PD mice were studied in depth. The catalepsy test was used to determine the changes in stiffness of the mice limbs, the rotarod test was used to determine the passive movement behavior changes, and the pole test was used to determine the adaptive exercise behavioral changes. Spontaneous behavioral changes were identified through the open field test and walking track analysis, while neuromuscular tension changes were identified through the grip strength test. The coordination ability, muscle stiffness, and neuromuscular tension of Parkinson’s disease mice have been well studied. Moreover, this study further explored the specific mechanism underlying the therapeutic effect of Safranal on PD mice, and it showed that Safranal mainly exerted its therapeutic effect by inhibiting the expression of inflammatory factors of the NLRP3 inflammasome. These discoveries were innovative and provided new targets for the clinical treatment of Parkinson’s disease. However, due to experimental condition limitations, other pathophysiological changes of striatum such as autophagy, mitochondrial metabolism, ROS, et al. in Parkinson’s mice have not been studied, Moreover, whether Safranal exerted its effects through other relevant mechanisms has not been clarified. This will be the focus of our future research.

In conclusion, Safranal reduced movement disorders and increased motor coordination to exerted a therapeutic effect on Parkinson’s disease mouse, and the underlying mechanism was related to the inhibition of NLRP inflammasome activation.

## Data Availability

No datasets were generated or analysed during the current study.
